# A genome-wide CRISPR screen implicates plasma membrane asymmetry in exogenous C6-ceramide toxicity

**DOI:** 10.1242/bio.059695

**Published:** 2022-12-19

**Authors:** Siti Nur Sarah Morris, Kirandeep K. Deol, Mike Lange, James A. Olzmann

**Affiliations:** ^1^ Department of Molecular and Cell Biology, University of California, Berkeley, CA 94720, USA; ^2^ Department of Nutritional Sciences and Toxicology, University of California, Berkeley, CA 94720, USA; ^3^ Chan Zuckerberg Biohub, San Francisco, CA 94158, USA

**Keywords:** CRISPR, Screen, Ceramide, Lipid, Lipotoxicity, Membrane, Sphingolipid

## Abstract

The bioactive sphingolipid ceramide impacts diverse cellular processes (e.g. apoptosis and cell proliferation) through its effects on membrane dynamics and intracellular signaling pathways. The dysregulation of ceramide metabolism has been implicated in cancer evasion of apoptosis and targeting ceramide metabolism has potential therapeutic benefits as a strategy to kill cancer cells and slow tumor growth. However, the mechanisms of cancer cell resistance to ceramide-mediated cell death are vastly intertwined and incompletely understood. To shed light on this mystery, we performed a genome-wide CRISPR-Cas9 screen to systematically identify regulators of cancer resistance to the soluble short chain ceramide, C6 ceramide (C6-Cer). Our results reveal a complex landscape of genetic modifiers of C6-Cer toxicity, including genes associated with ceramide and sphingolipid metabolism, vesicular trafficking, and membrane biology. Furthermore, we find that loss of the phospholipid flippase subunit TMEM30A impairs the plasma membrane trafficking of its binding partner, the P4-type ATPase ATP11B, and depletion of TMEM30A or ATP11B disrupts plasma membrane asymmetry and promotes resistance to C6-Cer toxicity. Together, our findings provide a resource of genetic modifiers of C6-Cer toxicity and reveal an unexpected role of plasma membrane asymmetry in C6-Cer induced cell death.

## Teaser

**Summary:** A genome-wide CRISPR screen reveals a role for TMEM30A-regulated membrane asymmetry in governing cancer cell sensitivity to ceramide-induced cell death.

## INTRODUCTION

Ceramides are a class of bioactive sphingolipids that consist of a sphingoid base amide bound to a fatty acid [For reviews on ceramide see (Chaurasia and Summers, 2021; Morad and Cabot, 2013; Summers et al., 2019)]. The length and degree of saturation within the sphingoid base or the fatty acid determines the biological activities of the individual ceramides. Multiple pathways generate ceramide, including the breakdown of complex sphingolipids such as sphingomyelins by sphingomyelinases, the reacylation of sphingosine in the ceramide salvage pathway, and the *de novo* synthesis pathway involving the condensation of serine and palmitoyl-CoA.

Ceramide has been implicated in diverse cellular roles, including the regulation of apoptosis, autophagy, cell proliferation, immune response, and ER stress (Chaurasia and Summers, 2021; Morad and Cabot, 2013; Summers et al., 2019). These varied functions are mediated by ceramide's ability to regulate membrane dynamics (e.g. membrane fluidity through ceramide-enriched membrane platforms) (Grassmé et al., 2007) and intracellular signal transduction (e.g. its interactions with a host of effector proteins) (Stancevic and Kolesnick, 2010; Summers et al., 2019). For example, in the extrinsic apoptotic pathway, initiation by activation of death receptors belonging to the tumor necrosis factor (TNF) receptor superfamily triggers an increase in plasma membrane ceramide levels through the actions of sphingomyelinases (Pettus et al., 2002). The increased ceramide forms ceramide-enriched membrane platforms in the plasma membrane that prime death receptor clustering, facilitating the formation of death-inducing signaling complexes (Grassmé et al., 2003; Stancevic and Kolesnick, 2010). Ceramide's position in the apoptotic pathway makes it a desirable topic for cancer research as dysregulation of ceramide metabolism has been implicated in cancer resistance to apoptosis. For instance, reduced ceramide is associated with resistance to CD95 and TRAIL-induced apoptosis in a variety of cancer cell types (Voelkel-Johnson et al., 2005; White-Gilbertson et al., 2009). Moreover, therapeutics targeting ceramide anabolic enzymes sensitize cancer cells to apoptosis inducers. Similarly, raising the levels of ceramide through exogenous treatment with soluble short chain ceramides such as C6 ceramide (C6-Cer) can induce apoptosis in cancer cells and *in vivo* experiments delivering C6-Cer through nanoliposomes have been efficacious in pre-clinical mouse models of cancer (Flowers et al., 2012; Ji et al., 2010; Liu et al., 2010; Stover et al., 2005; Tagaram et al., 2011; Tran et al., 2008). These findings suggest that targeting ceramide metabolism could be an effective strategy for cancer treatment.

The incomplete understanding of the mechanisms that mediate cancer resistance or sensitivity to ceramide-related cell death remains an obstacle for further development of targeted therapeutics. Here, we employ genome-wide CRISPR-Cas9 screens to provide a resource of genetic modifiers that influence C6-Cer toxicity. Our findings reveal that a phospholipid flippase composed of TMEM30A and the P4-type ATPase ATP11B is required for C6-Cer toxicity, implicating membrane asymmetry as a key factor in C6-Cer-induced cell death.

## RESULTS

### Genome-wide CRISPR-Cas9 screen identifies regulators of C6-Cer toxicity

**Fig. 1.**
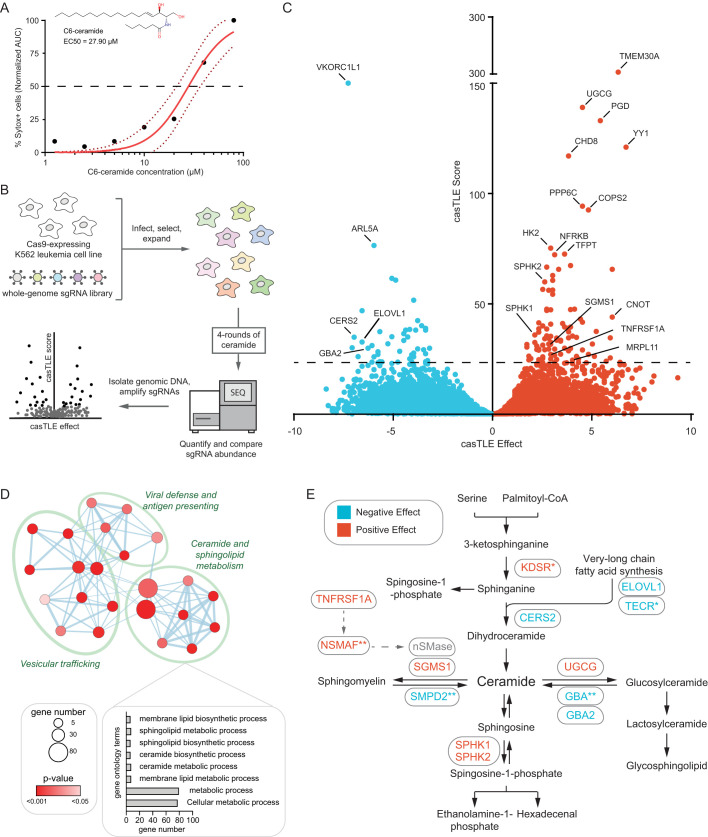
**Genome-wide CRISPR-Cas9 screen identifies genetic modifiers of exogenous ceramide toxicity.** (A) K562 cells were treated with the indicated concentrations of C6-Cer and percentage Sytox Green positive (Sytox+) cells was measured using an IncuCyte over 24 h. The structure of C6-Cer and the calculated EC50 are shown in the inset. Dotted lines indicate the 95% confidence intervals for the fitted curves. (B) Schematic of the CRISPR-Cas9 screening strategy. (C) Gene effect and gene score were calculated for individual genes analyzed in the CRISPR-Cas9 screen using the CasTLE. (D) Network map of enriched GO terms. Node size indicates gene number associated with a GO term and color indicates *P*-value. (E) Ceramide metabolic network indicating genes identified in the screen and whether knockout has a negative (blue) or positive (red) effect on cell survival in response to sequential rounds of C6-Cer treatments.

K562 chronic myelogenous leukemia cells have been previously shown to be sensitive to C6-Cer induced cell death, providing a useful model to study mechanisms of ceramide toxicity (Morad et al., 2015; Nica et al., 2008). Consistent with previous findings, K562 cells were sensitive to C6-Cer induced cell death with an EC50 of 27.90 µM (Fig. 1A). To systematically identify genetic modifiers of exogenous ceramide toxicity, we performed a genome-wide CRISPR-Cas9 knockout (KO) screen (Fig. 1B). K562 cells expressing Cas9 were infected with an sgRNA library containing 212,821 sgRNAs targeting 20,500 protein-coding genes (∼10 sgRNAs/gene) along with several thousand control sgRNAs. The cells were then treated with a vehicle or a 50% lethal concentration of C6-Cer. Four rounds of C6-Cer treatments were performed (with time for recovery), allowing sgRNAs that confer protection to be enriched and sgRNAs that confer sensitization to be depleted, relative to the vehicle-treated controls. The frequencies of the sgRNAs in the untreated and C6-Cer-treated cell populations were then determined by deep sequencing, and the enriched and depleted genes identified using Cas9 high-throughput maximum likelihood estimator (casTLE) (Morgens et al., 2016, 2017). The screening procedure was performed in duplicate and the data from both sets of screens were combined into a volcano plot depicting the collective casTLE confidence scores versus the casTLE phenotype effect score (Fig. 1C). Employing a 1% false discovery rate (FDR), we identified 97 genes that significantly influence C6-Cer toxicity, including genes that when depleted result in resistance (64 genes) and sensitization (33 genes) to C6-Cer toxicity (Fig. 1C and Table S1).

Analysis of Gene Ontology (GO) pathway enrichments for the identified genetic modifiers revealed expected connections to ceramide and sphingolipid metabolism (Fig. 1D,E and Fig. S1A,B). C6-Cer resistance factors (i.e. cells were sensitized to C6-Cer toxicity when these genes were depleted) that are canonically involved in ceramide metabolism included ceramide synthase, CERS2; the very-long chain fatty acid synthesis enzymes, ELOVL1 and TECR; sphingomyelin phosphodiesterase, SMPD2; and glucoceramidases, GBA and GBA2 (Fig. 1E). C6-Cer sensitizing factors (i.e. cells were resistant to C6-Cer death when these genes were depleted) included ceramide glucosyltransferase, UGCG; the sphingosine kinases, SPHK1 and SPHK2; and sphingomyelin synthase, SGMS1 (Fig. 1E). Why the paradoxical depletion of genes associated with ceramide anabolism would result in resistance to C6-Cer toxicity is not immediately clear. It may be that these cell lines have adapted to higher levels of ceramides with increased protective mechanisms to suppress ceramide toxicity. In addition to genes directly involved in ceramide and sphingolipid metabolism, the TNF receptor TNFRSF1A (also known as TNFR1) and NSMAF were identified as sensitizing factors (Fig. 1E). The identification of ceramide and sphingolipid metabolic genes, as well as extrinsic apoptosis factors that act through ceramide, is consistent with the high performance of our CRISPR-Cas9 screening platform.

**Fig. 2.**
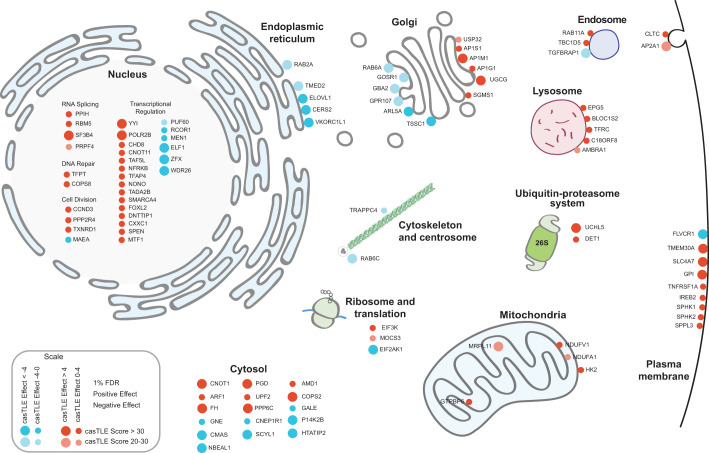
**Cell map of genetic modifiers of C6-Cer toxicity identified in CRISPR-Cas9 screens.** Cell diagram displaying 20 of the most significant hits detected in genome-wide screens for regulators of C6-Cer toxicity. Node size indicates casTLE score (i.e. confidence) and color indicates casTLE effect (i.e. phenotype), with the positive effect in red and negative effect in cyan.

The GO analysis also revealed significant enrichments in genes involved in vesicular trafficking and membrane biology (Fig. 1D and Fig. S1A,B). Consistent with this relationship, mapping genetic modifiers of C6-Cer toxicity onto a cell diagram identified a high number of genes within the secretory pathway. These included genes related to endoplasmic reticulum (ER) and Golgi trafficking as well as endocytic genes related to endosome and lysosome functions (Fig. 2). The connection with vesicular trafficking is in part because several of the enzymes involved in ceramide and sphingolipid metabolism localize to the plasma membrane and thus, secretory pathways (Fig. 2). However, many other genes involved in secretory protein trafficking were present, including Rab GTPases: Rab2A, Rab6A, Rab11a; the RAB GTPase activity proteins (RAB GAPs): TBC1D5 and ARF1; the cargo receptor, TMED2; the tSNARE protein, G0SR1; the EARP and GARP complex-interacting protein 1, TSSC1; the subunits of the clathrin-associated adaptor protein complex 1: AP1S1, AP1M1, AP1G1; and the clathrin-associated adaptor protein complex 2, AP2A1 (Fig. 2). Many genes in the cell map were also present in nuclear transcriptional pathways (Fig. 2), potentially reflecting the influence of multiple gene expression programs on ceramide and sphingolipid metabolism.

### Validation of select genetic modifiers of C6-Cer toxicity

**Fig. 3.**
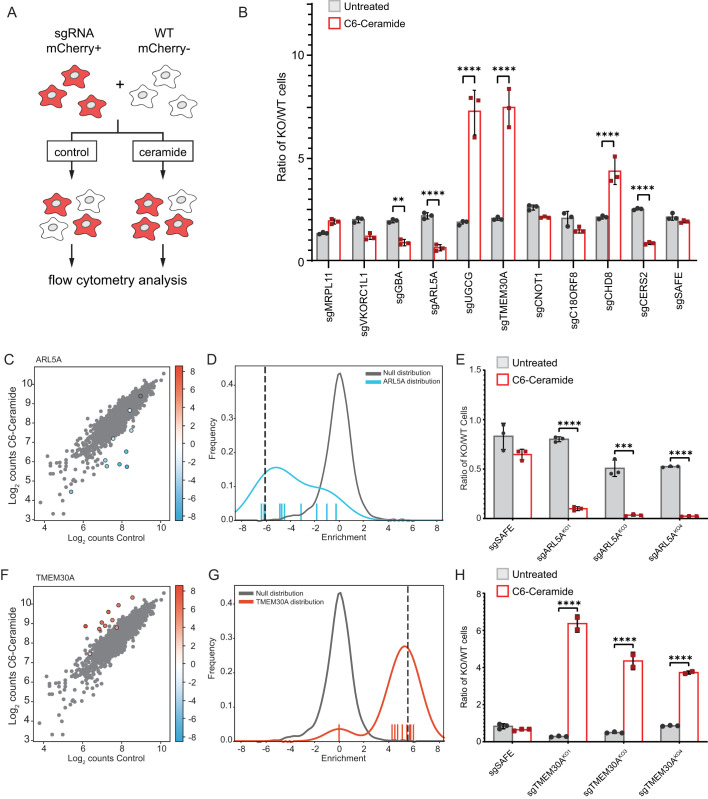
**Validation of select regulators of exogenous C6-Cer toxicity.** (A) Schematic of fluorescent competitive growth assay. (B) Ratio of KO/WT cells grown in the competitive growth assay depicted in panel A. Cells are untreated or treated with 30 µM C6-Cer for 24 h and allowed to recover prior to analysis. (C,D) Cloud plot and histogram indicating the disenriched sgRNAs targeting ARL5A following C6-Cer treatment in the CRISPR screen. (E) Competitive growth assay for analysis of three independent ARL5A KO lines. (F,G) Cloud plot and histogram indicating the enriched sgRNAs targeting TMEM30A following C6-Cer treatment in the CRISPR screen. (H) Competitive growth assay for analysis of three independent TMEM30A^KO^ lines.

We validated our screen results for 10 candidate regulatory genes using competitive growth assays. Our selection criteria of the genes to validate were based upon their cellular functions (e.g. known involvement in cellular trafficking and membrane homeostasis) and the strength of the casTLE confidence and effect scores (within the 1% false-discovery rate). In this assay (Fig. 3A), Cas9-expressing cell lines were infected with sgRNAs targeting candidate regulators of interest. Cells expressing the sgRNA plasmid co-express mCherry. These cells were combined in an equal ratio with a control line that does not express mCherry. This mixed cell population was treated with C6-Cer and then analyzed using flow cytometry to calculate the ratio of mCherry positive cells to mCherry negative cells. Cell lines expressing sgRNAs that confer resistance to C6-Cer death are proportionally higher in number than the control cells, while cell lines expressing sgRNAs that sensitize them to C6-Cer are less abundant than the control cells. Expression of sgRNAs targeting ceramide metabolic factors showed the anticipated effects based on our screen results, with depletion of CERS2 and GBA sensitizing cells to C6-Cer and depletion of UGCG promoting resistance to C6-Cer (Fig. 3B). In addition, sgRNAs targeting CHD8 and TMEM30A both resulted in strong resistance to C6-Cer toxicity and sgRNAs targeting ARL5 sensitized cells to C6-Cer (Fig. 3B). We did not observe a statistically significant effect of sgRNAs targeting MRPL11, VKORC1L1, CNOT1, or C18ORF8 (Fig. 3B). This difference may be due to insufficient depletion of the target or due to the difference in treatment paradigms – in the screen, four consecutive C6-Cer treatments were performed versus the single treatment during the competitive growth assay.

Among the panel of genes analyzed, sgRNAs targeting ARL5A and TMEM30A yielded the strongest sensitization and resistance to C6-Cer, respectively (Fig. 3B). KO of UDP-glucose ceramide glucosyltransferase (UGCG) also yielded strong resistance to C6-Cer. As UGCG is a gene that is known to be connected with ceramide metabolism, we chose to follow up on the more novel regulators – ARL5A and TMEM30A. Consistent with the high confidence identification of these candidate regulators, eight of the 10 ARL5a-targeting sgRNAs were depleted and 10 of the 10 TMEM30A-targeting sgRNAs were enriched following C6-Cer treatment in our screen (Fig. 3C,D,F,G). ARL5a is a small GTPase that may function in the recruitment of the GARP complex to the trans-Golgi network to regulate retrograde trafficking. TMEM30A is a subunit of phospholipid flippase complexes. It binds and facilitates the trafficking of several different P4-type ATPases to the plasma membrane, where they constitute functional lipid flippase complexes that regulate membrane asymmetry (Best et al., 2019; Hankins et al., 2015). To further validate these regulators of C6-Cer toxicity, we generated additional lines expressing unique sgRNAs targeting ARL5a and TMEM30A. Commercially available antibodies against ARL5a and TMEM30a proved unreliable, therefore, we verified our KO cell lines using qPCR and tracking of indels by decomposition (TIDE) (Brinkman et al., 2014). Three independent ARL5a-targeting sgRNAs sensitized cells to C6-Cer (Fig. 3E) and TMEM30A-targeting sgRNAs promoted resistance (Fig. 3H) to C6-Cer in the competitive growth assay, respectively. Furthermore, we found that two TMEM30A^KO^ cell lines exhibited enhanced resistance to C6-Cer induced cell death in an independent flow cytometry assay employing SYTOX Green, a membrane impermeable cell death marker (Fig. S2). Together, these data establish ARL5a and TMEM30A as novel regulators of C6-Cer toxicity.

### TMEM30A disrupts membrane asymmetry, but does not influence C6-Cer flipping or uptake

Phospholipid flippase complexes are composed of a catalytic α-subunit – which can be one of several P4-type ATPases – and the single accessory β-subunit TMEM30A (Best et al., 2019; Coleman and Molday, 2011; Shin and Takatsu, 2019; Timcenko et al., 2019). The association of TMEM30A with the P4-type ATPase enables the ER exit and trafficking of the complex to its final destination (e.g. plasma membrane) (Coleman and Molday, 2011; Shin and Takatsu, 2019). At the plasma membrane, the complex mediates selective phospholipid flipping to maintain the asymmetry of the plasma membrane interior and exterior leaflets (Best et al., 2019; Hankins et al., 2015). For example, these flippase complexes mediate the flipping of anionic phospholipids (e.g. phosphatidylserine, PS) from the outer to inner leaflet of the phospholipid bilayer (Hankins et al., 2015).

**Fig. 4.**
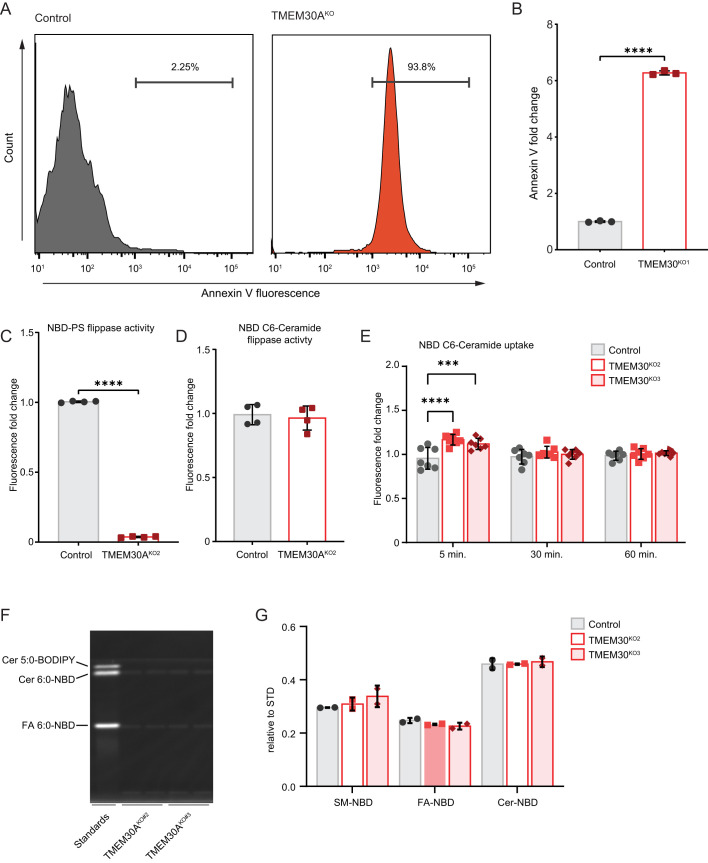
**Loss of TMEM30A disrupts phosphatidylserine flipping but not ceramide flipping nor uptake.** (A) Flow cytometry histograms of annexin V fluorescence in control and TMEM30A^KO1^ cells. (B) Quantification of annexin V fluorescence by flow cytometry (as in panel A). (C) Quantification of NBD-PS flippase activity by flow cytometry. (D) Quantification of NBD C6-Cer flippase activity by flow cytometry. (E,F,G) Quantification of NBD C6-Cer uptake by flow cytometry (E) and thin layer chromatography (F,G).

To examine the role of TMEM30A in membrane asymmetry in K562 cells, we ascertained the relative amount of PS in the outer leaflet via a flow cytometric assay using PS-binding Annexin V conjugated to FITC. TMEM30A^KO^ cell lines showed a drastic increase in Annexin V staining, with >90% of the cells being Annexin V positive and the population exhibiting an ∼6-fold increase in Annexin V staining (Fig. 4A,B). Dead cells were removed from this analysis by staining with SYTOX-Deep Red, ensuring that the Annexin V staining is not due to dead cells. To directly measure PS flippase activity, we incubated cells with fluorescently-tagged nitrobenzoxadiazole (NBD) PS and then used BSA to remove any NBD-PS remaining in the outer PM leaflet. The fluorescence, which represents PS that has flipped into the cytoplasmic leaflet, can then be measured by flow cytometry. As expected, TMEM30A^KO^ cells exhibited a significant impairment in PS flipping (Fig. 4C).

Ceramide is thought to spontaneously flip between bilayer membrane leaflets, an action independent of a protein-based transporter or flippase. We considered the possibility that the altered composition of the TMEM30A^KO^ inner and outer plasma membrane leaflets could influence C6-Cer flipping and cellular uptake. Using NBD C6-Cer, we performed a similar cell-based lipid flipping assay. In contrast to NBD-PS (Fig. 4C), NBD C6-Cer flipping was unaffected in the TMEM30A^KO^ cells (Fig. 4D). We also examined longer incubation times to determine if NBD C6-Cer uptake into cells is affected. Although there was a small, statistically significant increase in NBD C6-Cer fluorescence in the TMEM30A^KO^ cells at 5 min, there was no difference at 30 or 60 min (Fig. 4E). To validate our ceramide uptake assay, we also measured NBD C6-Cer uptake using thin layer chromatography. In addition to the NBD C6-Cer, we observed a band corresponding to the NBD-labelled fatty acid, indicating metabolism of the NBD C6-Cer following cellular uptake (Fig. 4F,G). Loss of TMEM30A had no effect on the levels of NBD C6-Cer or the NBD-conjugated fatty acid (Fig. 4F,G). Together, our data indicate that although TMEM30A^KO^ cells exhibit reduced PS flipping and altered membrane asymmetry, there is no defect in C6-Cer flipping or uptake.

### TMEM30A trafficking of ATP11B to the plasma membrane impacts C6-Cer toxicity

**Fig. 5.**
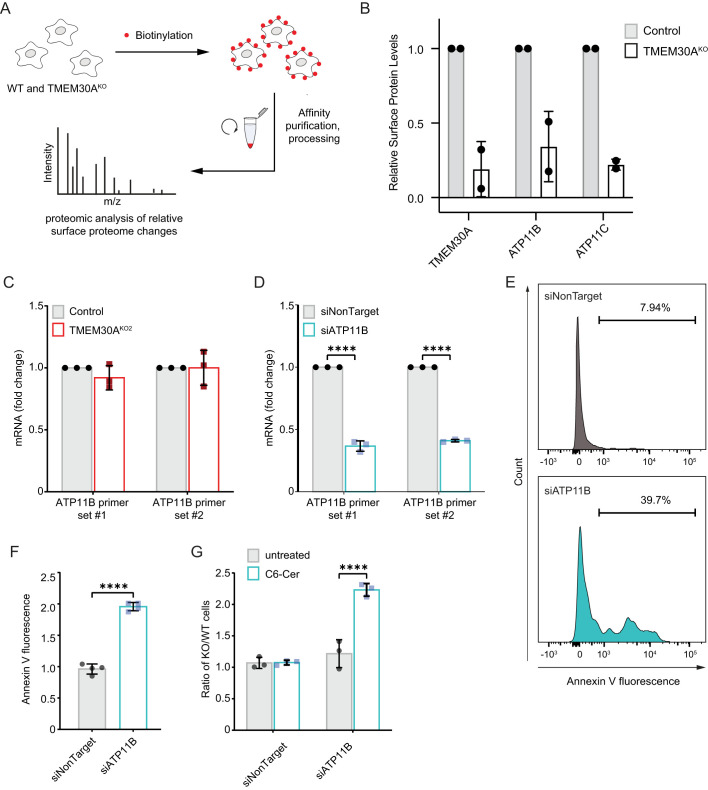
**Impaired plasma membrane trafficking of ATP11B impacts PS flipping and exogenous C6-Cer toxicity.** (A) Schematic of surface proteomics approach. (B) Quantification of the relative surface proteome levels for TMEM30A, ATP11B, and ATP11C in control and TMEM30A^KO2^ cells. (C) Quantification of ATP11B mRNA by RT-PCR in control and TMEM30A^KO2^ cells. (D) Quantification of ATP11B mRNA by RT-PCR in cells transfected with siRNAs, siNonTarget and siATP11B. (E) Flow cytometry histograms of annexin V fluorescence following transfection with siNonTarget and siATP11B. (F) Quantification of annexin V fluorescence following transfection with siNonTarget and siATP11B (as in panel D). (G) C6-Cer competitive growth assay of cells transfected with the indicated siRNA against an untransfected control cell line.

The canonical role of TMEM30A is as a subunit of phospholipid flippase complexes that is required both for the trafficking of the flippase complex to the plasma membrane. Indeed, we observed alterations in TMEM30A^KO^ cells PS flippase activity and plasma membrane composition (Fig. 4). These results raise the possibility that the alteration in plasma membrane asymmetry is responsible for the C6-Cer resistance of TMEM30A^KO^ cells. No P4-type ATPases were identified in the 1% FDR cut-off of our genetic screen, possibly reflecting compensation due to the overlapping functions of these proteins. To identify P4-type ATPases that exhibit impaired trafficking in the TMEM30A^KO^ cells, we implemented a proteomics workflow to examine changes in the surface proteome (Fig. 5A). We identified 3442 proteins, including many expected plasma membrane proteins (Table S2). As anticipated, TMEM30A was reduced in the TMEM30A^KO^ cell lines (Fig. 5B). ATP11B and ATP11C, two P4-type ATPase that associate with TMEM30A, exhibited reduced plasma membrane abundance in multiple TMEM30A^KO^ cell lines (Fig. 5B). We focused on ATP11B because of its higher abundance, based upon the number of identified spectral counts (Table S2). The reduction in ATP11B levels was not due to altered ATP11B gene expression in the TMEM30A^KO^ cells (Fig. 5C).

To examine the functional role of ATP11B, we depleted ATP11B using siRNAs (Fig. 5D). ATP11B depletion resulted in an increase in the percentage of Annexin V positive cells and fluorescence (Fig. 5E,F), consistent with a role for ATP11B in the maintenance of membrane asymmetry in K562 cells. Similar to the loss of TMEM30A, the depletion of ATP11B also resulted in resistance to C6-Cer toxicity (Fig. 5G). The effects of ATP11B on membrane asymmetry and C6-Cer resistance were not as large as in the TMEM30A^KO^ cells, possibly due to the partial depletion of ATP11B or the contribution of additional P4-type ATPases such as ATP11C, which also exhibited reduced plasma membrane levels in the TMEM30A^KO^ cells. Together, these results suggest that TMEM30A influences C6-Cer resistance, at least in part, by promoting ATP11B trafficking and plasma membrane asymmetry.

## DISCUSSION

Ceramide plays established roles in apoptosis through its regulation of key signaling events (Morad and Cabot, 2013). The mechanisms by which cancer cells evade ceramide-induced cell death remain incompletely understood. We employed a genome wide CRISPR-Cas9 screen to provide a resource of high confidence genetic modifiers of C6-Cer induced cell death in K562 cells and to reveal a role for plasma membrane symmetry in governing the sensitivity of cells to C6-Cer toxicity.

Our genetic screen identified a host of genes from diverse functional categories, these included enrichments in ceramide and sphingolipid metabolism, membrane biology, vesicular trafficking, and transcription. We provide initial validation of several candidate regulators, including CERS2, GBA, UGCG, CHD8, ARL5, and TMEM30A. Further studies will be required to understand the mechanisms that mediate their role in C6-Cer induced cell death. It is notable that we identified the TNF receptor TNFRSF1A (also known as TNFR1) and NSMAF in our screen using C6-Cer. In the extrinsic apoptotic pathway, TNF binding to TNF receptors leads to ceramide generation whilst NSMAF couples the TNF receptor to neutral sphingomyelinase to produce ceramide from sphingomyelin catabolism. The identification of TNFRSF1A suggests that our screen may detect factors that will influence endogenous ceramide apoptotic signaling in addition to the cell death triggered by the more artificial exogenous C6-Cer treatment.

One of the most interesting discoveries was that the loss of TMEM30A results in resistance to C6-Cer toxicity. TMEM30A is a subunit in phospholipid flippase complexes (Hankins et al., 2015). It binds several P4-type ATPases and is integral to proper ATPase trafficking and function (Coleman and Molday, 2011). Our proteomic analyses of the surface proteome in TMEM30A^KO^ cells revealed a loss of ATP11B, reaffirming the requirement of TMEM30A for trafficking of P4-type ATPases. RNAi-mediated depletion of ATP11B phenocopied the loss of TMEM30A, resulting in the localization of PS to the outer leaflet of the plasma membrane and in resistance to C6-Cer toxicity. This result supports the model that loss of plasma membrane asymmetry is responsible for the C6-Cer resistance phenotype in the TMEM30A^KO^ cells. The effects on PS exposure and C6-Cer resistance were more modest in the ATP11B depleted cells than in the TMEM30A^KO^ cells and it remains possible that other P4-type ATPases also contribute to the observed phenotypes. Ceramide is known to spontaneously flip within the bilayer independently of a protein-based transporter. We did not observe any defect in NBD-C6-Cer flipping or uptake, suggesting that the resistance phenotype is not simply due to a reduction in C6-Cer access to the interior of the cell. However, it should be noted that NBD-C6-Cer has distinct molecular properties relative to C6-Cer. The addition of the NBD group increases the size of the molecule and, due to the polar nature of NBD, may cause the acyl chain to loop back towards the membrane–water interface. Further studies using lipidomics approaches will be necessary to definitively determine whether uptake and/or flipping of ceramide are affected in the TMEM30A^KO^ cells.

The plasma membrane is a complex combination of phospholipids, sterols, glycolipids, and proteins (Harayama and Riezman, 2018). In addition, the composition of the inner (cytoplasmic) and outer (exoplasmic) leaflets of the plasma membrane differ (Doktorova et al., 2020). Employing energy generated from ATP hydrolysis, lipid flippases transfer lipids from the outer leaflet to the inner leaflet (Hankins et al., 2015). Flippases act together with floppases–which transfer lipids from the inner to outer leaflets, and scramblases, which mediate bidirectional lipid transport, to dynamically control transbilayer lipid compositions (Hankins et al., 2015). Elegant lipidomics studies indicate that the outer leaflet of the plasma membrane is primarily composed of phosphatidylcholine and sphingolipids, which tend to pack tightly and contribute to a more highly ordered and rigid membrane (Doktorova et al., 2020). The inner leaflet of the plasma membrane is enriched in charged lipids, including PS, phosphatidylethanolamine, phosphatidylinositol, and overall contains more highly unsaturated fatty acids (Doktorova et al., 2020). The canonical enrichment of charged lipids within the inner leaflet contributes to the electrostatic potential across the bilayer (Hankins et al., 2015; Harayama and Riezman, 2018). This influences the insertion and folding of integral membrane proteins as well as the association of peripheral proteins through polybasic stretches (Harayama and Riezman, 2018). As anticipated, we found that PS aberrantly localizes to the outer leaflet of the plasma membrane in the TMEM30A^KO^ cells. The transbilayer distribution of other lipids, such as sphingolipids, in the TMEM30A^KO^ is not known but broader alterations in the composition of plasma membrane inner and outer leaflets may contribute to our observed phenotypes.

While our results implicate plasma membrane asymmetry in cellular sensitivity to C6-Cer toxicity, the exact mechanism is not clear and will require additional studies. Alterations in membrane asymmetry may influence sphingolipid and ceramide metabolic pathways, which would result in changes in intracellular ceramide levels that would affect the formation rate of ceramide-enriched platforms and death-induced signaling complexes. In addition, the altered plasma membrane asymmetry likely influences integral membrane protein topologies and charge-based recruitment of peripheral proteins to the plasma membrane. Plasma membrane asymmetry may also influence the ability of ceramide to form channels that permeabilize membranes, though this activity of ceramide is controversial.

Together, our study provides a global overview of the genetic landscape that governs C6-Cer toxicity. Moreover, our findings implicate plasma membrane asymmetry as a key factor in the cellular sensitivity to C6-Cer, setting the stage for future studies examining the connection between specific plasma membrane lipids and ceramide metabolism, plasma membrane physical properties, and cell death pathways.

## MATERIALS AND METHODS

### Cell lines and culture conditions

HEK293T was obtained from UC Berkeley's cell culture facility. K562 and K562-Cas9-BFP cells were a generous donation from Professor Ron Kopito (Stanford University, CA, USA). HEK293T was maintained in DMEM containing 4.5 g/L glucose and L-glutamine (Corning, 10-017). K562 cells and their derivatives were maintained in RPMI-1640 with L-glutamine (Corning, 10-040-CM). All media was supplemented with 10% fetal bovine serum (Gemini Bio Products) and penicillin (10,000 I.U ml^−1^). Cell lines were maintained at 37°C with 5% CO_2_. Cell line identifies were not authenticated. Regular screening for mycoplasma contamination.

### EC_50_ death assay

Cell death was assayed via the Essen IncuCyte Live-Cell imaging system (Sartorius). Ten thousand K562 cells per well were seeded in black 96-well plates (Corning, 3904). Media containing a final concentration of 15 nM SYTOX-Green Dead Cell Stain (Invitrogen, S34860) and C6-Ceramide (d18:1/6:0) (Enzo, BML-SL110) at various concentrations was added to produce a final cell density of 500,000 cells/ml. Plates were carefully transferred to the IncuCyte system (kept at 37°C with 5% CO_2_) and imaged for 24 h. Three images per well were captured in the green and brightfield channels every hour for the treatment period. The Sartorius image analysis software outputs the number of green objects (SYTOX-Green positive, i.e. dead cells) as well as the total number of objects (brightfield segmentation). For each C6-Cer concentration, the ratio of dead objects over total objects was plotted over the 24 h imaging cycle. From this, Prism (Graphpad) was used to calculate the area under the curve (AUC), plotted as function of C6-Cer dosage and mathematically determine the EC_50_.

### CRISPR-Cas9 synthetic lethal screen

The screen was performed as previously described. In brief, the Bassik Human CRISPR Knockout Library (Addgene, 101926-101934) is separated into nine sublibraries comprising a total of 225,171 elements and targeting approximately 20,500 genes (10 sgRNAs per target). To generate the lentiviral library pool, each sublibrary pool was co-transfected with third-generation lentiviral packaging plasmids [pVSV-G/MD2.G (Addgene, 12259), pRSV-Rev (Addgene, 12253) and pMDLg (Addgene, 12251)] into low-passage HEK293T cells. Lentiviral-containing media was collected at 48 h and at 72 h post-transfection. These media pools were then combined and filtered via 0.45-micron cartridge. The resultant lentiviral media was used to infect approximately 2.0×10^8^ K562-Cas9 cells for 72 h. After this infection period, the cells were spun down at 500×***g*** for 5 min and viral-laden media was removed. These cells then underwent 5 µg/ml puromycin dihydrochloride (Gibco, A1113803) selection until the population was over 97% mCherry positive. Cells then recovered in puromycin-free media until a total of 2.5×10^8^ (∼1000-fold total library elements). This pool was then split and treated with either ethanol (EtOH) or 30 µM C6-Cer for 24 h. The treatment period was followed by a 48 h recovery and subsequently, the treatment cycle was repeated thrice more. Each subsequent cycle required a slight uptick in C6-Cer concentration as the pools experienced rapid resistance to death and thus the drug concentration was raised to achieve ∼50% death. The dosage for cycle 2 and upwards is as follows: 32.5 µM, 35 µM and finally, 40 µM. Throughout the screen, K562 cells are maintained at 500,000 cells/ml.

After the last bout of treatment, cells were twice-washed with PBS, pelleted into numbers approximating 250-fold of total library elements and stored at −80°C. Genomic DNA was extracted using the QIAmp DNA Blood Maxi Kit (Qiagen) according to the manufacturer's directions. This genomic DNA underwent two rounds of PCR to first amplify sgRNA sequences and then index them using Illumina TruSeq LT adaptor sequences.

PCR amplicons from each sample were pooled into a 1:1 ratio (EtOH:C6-Cer) based on concentrations determined by Qubit Fluorometric Quantification (Invitrogen). Deep sequencing was done on an Illumina NextSeq instrument at the Oklahoma Medical Research Foundation. Sequence reads were trimmed, then aligned using Bowtie with zero mismatches tolerated. Enrichment, confidence scores and *P*-values were calculated using the casTLE Python code as previously described.

### Generation of CRISPR-Cas9 genome-edited cell lines

KO cell lines were generated the same way as the lentiviral portion of the CRISPR-Cas9 screen. Cas9 cutting functionality was validated via infecting K562-Cas9 with a self-cutting mCherry plasmid (a kind gift from Professor Michael Bassik, Stanford University, CA, USA) that expresses mCherry as well as a sgRNA sequence targeting said gene.

sgRNA sequences selected from the CRISPR KO screen were used to create the individual transfection plasmids. All guide sequences were inserted into pMCB320 between the BstXI and BlpI sites as previously described.

**Table:**
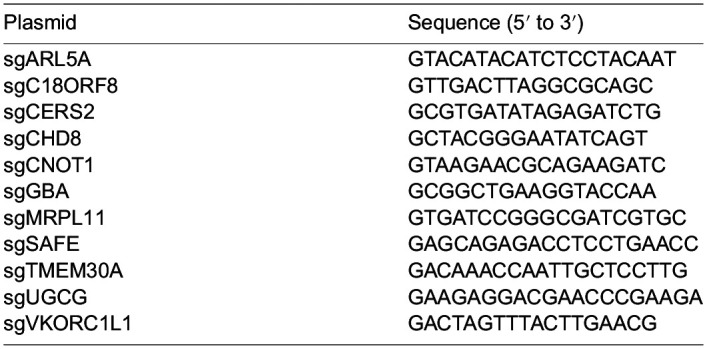


Single clones were isolated from the sgTMEM30A polyclonal line to create TMEM30A^KO1^ and TMEM30A^KO2^ via serial dilution. Serial dilution was also employed to form clonal lines of sgARL5A to create ARL5A^KO1^ and ARL5A^KO2^. The sgRNA sequences to the additional knockout lines are as follows:

**Table:**
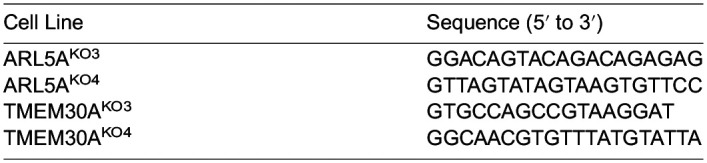


### Competitive growth assay

WT cells (self-cut mCherry, no mCherry emission) and KO cells (mCherry positive) were seeded in a 1:1 ratio with a final density of 500,000 cells/ml into a 96-well round-bottomed plate. Wells were treated with either vehicle (EtOH, untreated) or C6-Ceramide for 24 h. Cells were then spun down, media aspirated and then replenished with media containing 15 nM SYTOX-Green. The plate was assayed via flow cytometry on a BD LSRFortessa in the UC Berkeley Flow Cytometry Facility, CA, USA; dead cells were gated out (positive SYTOX-Green) and live cells were analyzed to discover the percentages of mCherry +/- cells. Two-way ANOVA was calculated on Prism.

### Annexin V assay

Assay was adapted from manufacturer directions of the FITC Annexin V Apoptosis Detection Kit (BD Pharmingen, 556547). In brief, K562 cells were collected from cultures that were 98% live (determined by Trypan Blue staining). Samples were incubated with SYTOX-Deep Red Live/Dead Fluorescent Dye (Invitrogen, S11380) for 15 min at 37°C, then washed twice with DPBS (Gibco, 14190-144) before proceeding with Annexin V-FITC incubation. Samples were analyzed by flow cytometry; dead cells (i.e. SYTOX-Deep Red positive) were gated out and the FITC spectra measured of the remaining population.

### Flippase assay

NBD C6-Ceramide (Cayman, 62527) and NBD PS (Avanti, 810194C) were prepared as previously described (Kay et al., 2012). In brief, compounds were dried under N2 and resuspended in methanol. Incorporation of lipids is modified from methods described (Goñi et al., 2014; Sandhoff et al., 2018). Compounds are pre-incubated in 4 mg/ml BSA (Sigma-Aldrich, A8806) in H_2_O under constant rotation at 300 rpm for 30 min at 37°C. K562 cells were collected via centrifugation, washed and equilibrated at 15°C for 30 min in Hank's balanced salt solution (HBSS) containing 1 g/L glucose (Gibco, 14025-092) at a concentration of 500,000 cells/ml. After cell equilibration, the incubated compounds were added to the cell suspension to the required lipid concentration. This mixture was incubated at 15°C for 20 min with constant rotational speed of 300 rpm. At the end of the timepoint, NBD conjugated compounds were mixed with equal volume of ice-cold 5 mg/ml BSA in HBSS to extract the lipids incorporated into the exoplasmic PM leaflet. Cells were then analyzed by flow cytometry to find the geometric mean of FITC emission. Replicates were averaged and normalized to control values.

### Uptake assay

Uptake assay is adapted from the flippase method. Samples were plated in a 96-well round bottom plate to a final concentration of 500,000 cells/ml with lipid incorporation occurring in regular media. Samples were incubated at 37°C at 5% CO_2_ for the designated timepoints. At the end of each timepoint, cells were washed thrice with DPBS and assayed by flow cytometry. Replicates were averaged and normalized to control values.

### High performance thin-layer chromatography

Cells were isolated and washed twice with PBS. Cells were then pelleted at 500×***g*** for 5 min, the supernatant was removed, and cell pellets were stored at −80°C. Before extraction, cell pellets were thawed on ice for 30 min and resuspended in 50 μl PBS. Lipids were extracted by adding 1250 μl tert-butyl methyl ether (HPLC grade, Sigma-Aldrich) and 375 μl methanol (HPLC grade, Fisher Scientific, both containing 0.1% (w/v) 2,6-Di-tert-butyl-4-methylphenol (GC grade, Sigma-Aldrich). The mixture was incubated on an orbital mixer for 1 h (room temperature, 32 rpm). To induce phase separation, 315 μl water containing 0.1% (w/v) 2,6-Di-tert-butyl-4-methylphenol (GC grade, Sigma-Aldrich) was added, and the mixture was incubated on an orbital mixer for 10 min (room temperature, 32 rpm). Samples were centrifuged (room temperature, 10 min, 17,000×g). Upper organic phase was collected and subsequently dried *in vacuo* (Eppendorf concentrator 5301, 1 ppm).

The lipid extract was dissolved in 50 μl chloroform/methanol (2:1, v/v) and 5 μl of each extract were loaded on silica coated, glass-bottomed plates (HPTLC silica gel 60, 10×10 cm, Merck). Pure standards of FA 6:0-NBD (Cayman Chemical), Cer 18:1;O2/6:0-NBD (Cayman Chemical) and Cer 18:1;O2/5:0-BODIPY (Cayman Chemical) were loaded as 0.1 nmol aliquots on the same plates. Lipids were separated using a solvent mixture of triethylamine/chloroform/ethanol/water (5:5:5:1, v/v) (all solvents HPLC grade, Sigma-Aldrich) as mobile phase in a solvent vapour saturated twin trough chamber (CAMAG, Switzerland). Separated lipids were imaged directly on glass-backed TLC plates using a Gel Doc EZ System (BioRad, USA) in combination with the UV Tray filter (BioRad). Densitometric quantification of lanes was performed using Image Lab Software Version 5.2.1 (BioRad)

### Surface biotinylation assay and proteomics

All buffers were filter sterilized prior to use. Each sample required 24 million cells that were pelted and washed twice with PBS. 10 mM of freshly prepared EZ-Link™ Sulfo-NHS-SS-Biotin (Thermo Fisher Scientific, A39258) in PBS and added to the cell mixture at 80 µl of 10 mM NHS-SS-Biotin per ml of sample volume. Samples were incubated by rocking at room temperature for 30 min. After incubation, cells were pelleted at 300 ***g***-force for 3 min, excess biotin discarded and then washed twice with ice-cold TBS.

Proteins were collected from cells via lysing with dissolved Pierce™ Protease Inhibitor Mini Tablets, EDTA-free (Thermo Fisher Scientific, A32955) in RIPA buffer (Thermo Fisher Scientific, 89901). Samples were sonicated for 15 s at 15% amplitude, followed by vortexing for 10 s every 10 min for 30 min. This was followed by a 5 min centrifugation at 15,000 ***g***-force and supernatant.

Enrichment of biotinylated proteins proceeded via an overnight incubation at 4°C on NeutrAvidin resin (Thermo Fisher Scientific, 53150) at a ratio of 10 µl of beads per 100 µg protein. After incubation, samples were centrifuged at 700 ***g***-force for 2 min at 4°C. Pellets then underwent a series of wash steps: twice with lysis buffer, thrice with ultrapure H_2_O and finally, twice with 50 mM ammonium bicarbonate (ABC), pH 8.0. A portion of clarified proteins were assessed for biotinylation efficiency via western blotting with a streptavidin secondary antibody (Li-Cor, 32230). Biotinylated proteins were eluted from resin using 10 mM dithiothreitol (Thermo Fisher Scientific, R0861) dissolved in 50 mM ABC by end-over-end rotation at room temperature for 30 min. Sample was centrifuged at 700 ***g***-force for 2 min and supernatant collected.

Sample preparation for mass spectrometry required the addition of 25 µl of freshly prepared 55 mM iodoacetamide (dissolved in 50 mM ABC) with a requisite 30 min incubation at room temperature in the dark. After the time interval, we added ice-cold acetone to mix overnight at −20°C. Samples were then centrifuged at 15,000 ***g***-force for 10 min, and then decanted for 30 min at room temperature to precipitate proteins.

Precipitated proteins were resuspended in 25 mM ammonium bicarbonate, digested overnight using 1 µg of trypsin (Promega, V5113) at 37°C. Samples were acidified with 10% v/v of trifluoroacetic acid, desalted using C18-stage tips, and dried. For MS analysis, peptides were resuspended in 1% formic acid and separated on an Easy nLC 1000 UHPLC equipped with a 15 cm nanoLC column. Using a flow rate of 300 nl/min, the linear gradient was 5% to 35% over B for 90 min, 35% to 95% over B for 5 min, and 95% hold over B for 15 min (solvent A: 0.1% formic acid (FA) in water, solvent B: 0.1% FA in ACN). The table indicates ley mass spectrometer parameters.

**Table:**
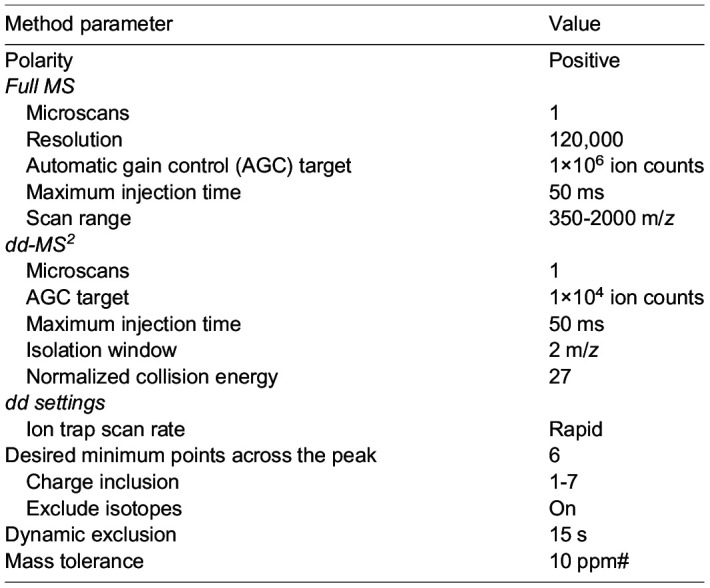


Peptide identities and relative abundances were determined using Proteome Discoverer 2.4. Ion chromatograms were extracted using Xcalibur Qual Browser for each peptide of interest with a mass tolerance of 0.5 Da. We thank Dr. Steve Eyles (University of Massachusetts Amherst, MA, USA RRID: SCR-019063) for assistance with high-resolution MS acquired on an Orbitrap Fusion mass spectrometer (National Institutes of Health grant: 1S10OD010645-01A1).

### siRNA knockdown of ATP11B

sgSAFE cells were transfected with either siATP11B (Horizon, L-023660-00-0005) or siNon-targeting control (Horizon, D-001810-10-05) using Lipofectamine RNAiMax transfection reagent (Thermo Fisher Scientific, 13778) according to manufacturer directions. Incubation proceeded for 48 h, whereupon samples were washed and divvied into three populations: two for subsequent competitive growth assays and Annexin V studies, with the last assessed for KO efficiency via RT-qPCR. K562 self-cutting mCherry cells were also transfected with siNon-targeting control to serve as the mCherry population in our competitive growth assay.

### RT-qPCR

Total RNA of cell samples was extracted using the Monarch Total RNA kit (NEB, T2010S) and then reverse transcribed with iScript cDNA Synthesis kit (Biorad, 1708890). cDNA was measured using a CFX96 Touch Deep Well Real-Time PCR system (Biorad) with the probe-based PrimeTime Gene Expression Master Mix (IDT, 1055770). Fold changes in cDNA were determined using the ΔΔC_t_ method normalized to PPIA cDNA. Primer pairs were purchased from IDT's predesigned PrimeTime Standard qPCR assay; sequences are as follows:

**Table:**
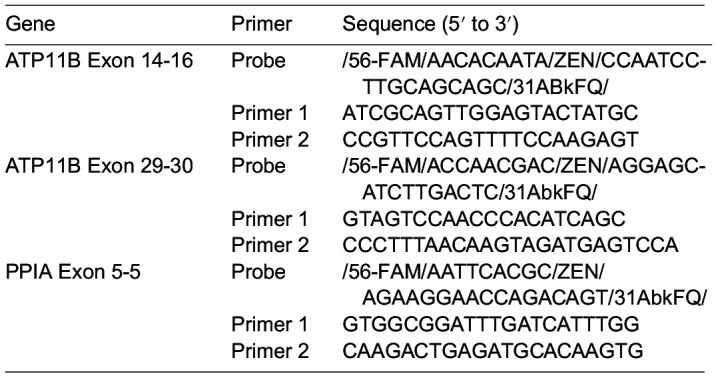


### Cell counts, statistical analysis and reproducibility

Unless otherwise indicated, cell numbers and concentrations were derived from live cell counts (determined by Trypan Blue staining). Experimental populations were collected from growing pools that exhibited ≥97% live. Additionally, unless otherwise indicated, experiments were done in triplicate (*n*=3) with statistical significance calculated in Prism using a two-tailed unpaired Students’ *t*-test (*P*-value ≤0.0332 *, ≤0.0021 **, ≤0.0002 ***, <0.0001 ****).

## Supplementary Material

10.1242/biolopen.059695_sup1 Supplementary information
