# Supplementary material for: A genome-wide CRISPR screen implicates plasma membrane asymmetry in exogenous C6-ceramide toxicity
Source: Biol Open. 2022 Dec 19;11(12):bio059695. doi: 10.1242/bio.059695 (PMC9793866; doi:10.1242/bio.059695)
Supplement: Supplementary information [file biolopen-11-059695-s1.pdf]

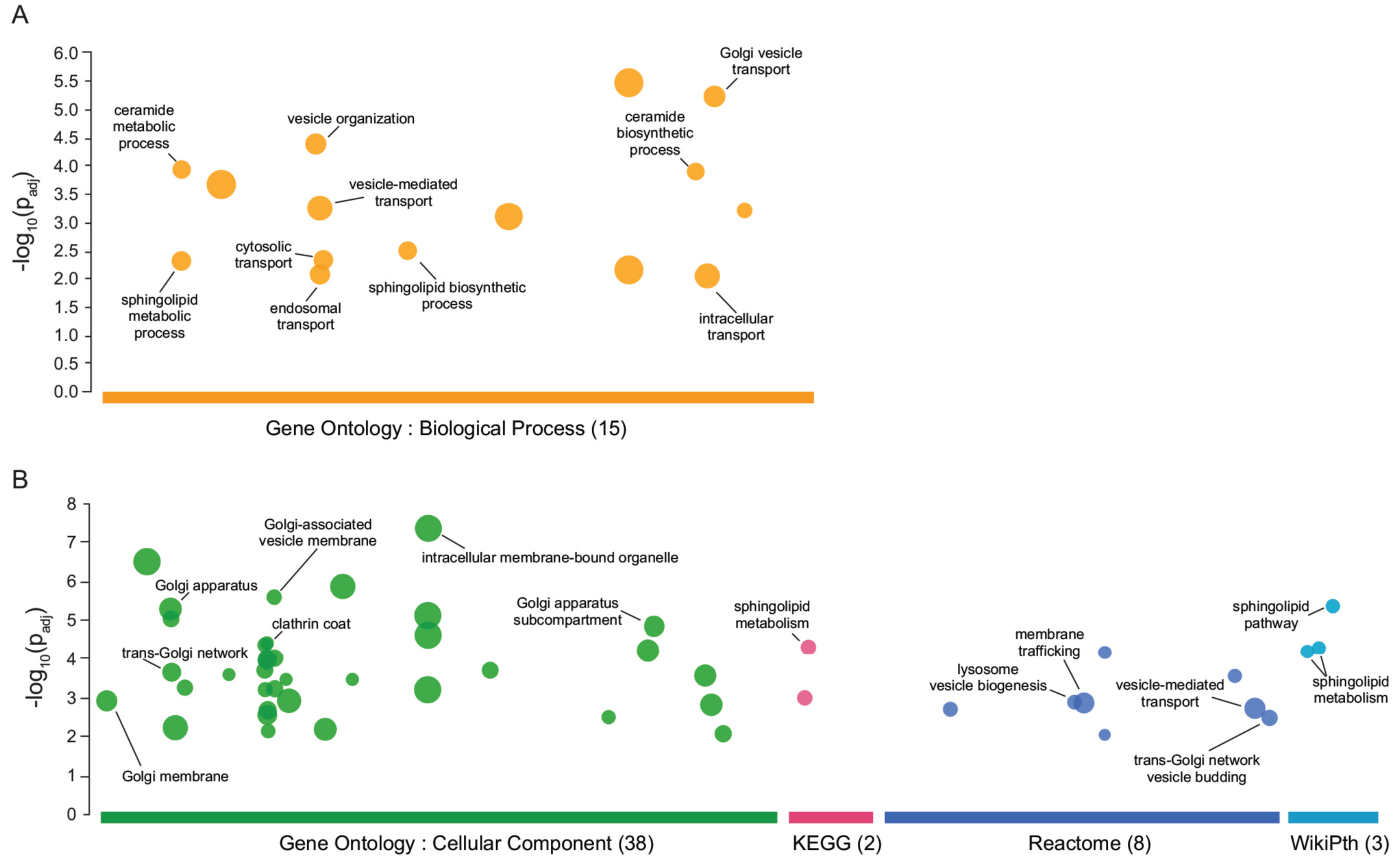

Fig. S1. Analysis of C6-Cer genetic modifiers.

Significantly enriched and disenriched genes from the genome-wide C6-Cer CRISPR screen were analyzed using g:Profiler to identify functional relationships.

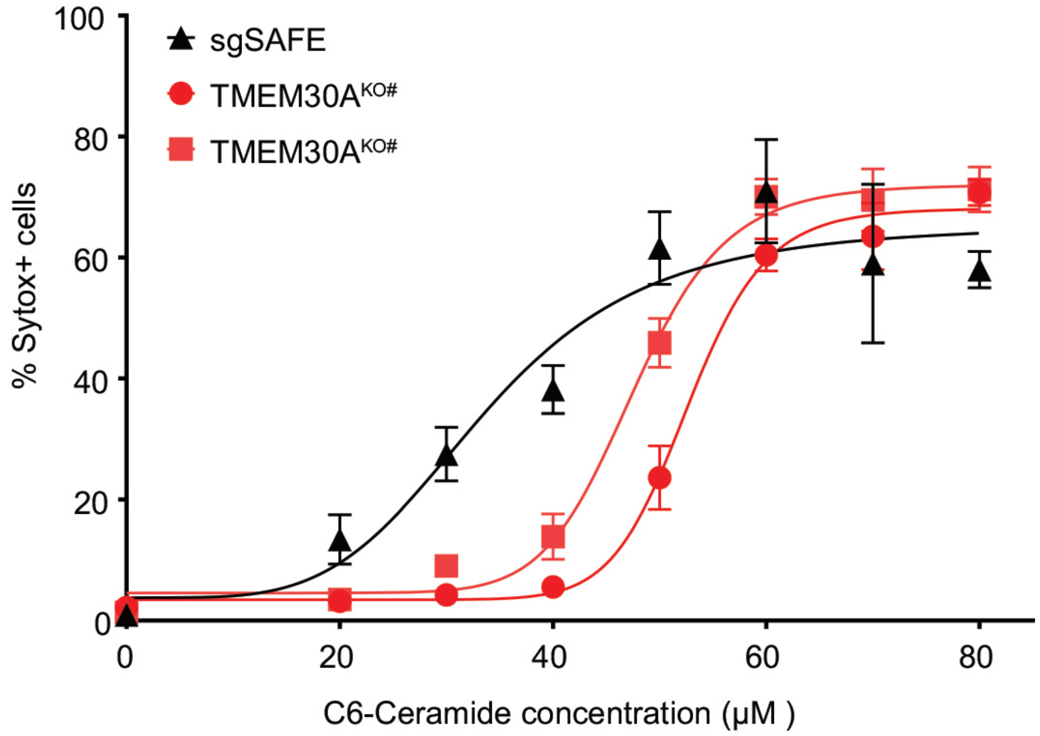

Fig. S2. Loss of TMEM30A sensitizes cells to exogenous C6-Cer-induced cell death.

K562 cells were treated with the indicated concentrations of C6-Cer for 24 hr and the percentage of dead cells (Sytox+) quantified using flow cytometry.

Table S1. CRISPR-Cas9 screen data.

Click here to download Table S1

Table S2. Surface proteome analysis.

Click here to download Table S2
